# Phycocyanin Additives Regulate Bacterial Community Structure and Antioxidant Activity of Alfalfa Silage

**DOI:** 10.3390/microorganisms12122517

**Published:** 2024-12-06

**Authors:** Xiangdong Liu, Yichao Liu, Mingjian Liu, Jing Xing, Yuxuan Wang, Panjie Sheng, Gentu Ge, Yushan Jia, Zhijun Wang

**Affiliations:** Key Laboratory of Forage Cultivation, Processing and High Efficient Utilization, Ministry of Agriculture, Key Laboratory of Grassland Resources, Ministry of Education, College of Grassland Science, Inner Mongolia Agricultural University, Hohhot 010019, China; 18847788933@163.com (X.L.); gegentu@163.com (G.G.)

**Keywords:** phycocyanin, alfalfa silage, antioxidant capacity, fermentation quality

## Abstract

Phycocyanin is a water-soluble pigment protein extracted from prokaryotes such as cyanobacteria and has strong antioxidant activity. As a silage additive, it is expected to enhance the antioxidant activity and fermentation quality of alfalfa silage. This study revealed the effects of different proportions of phycocyanin (1%, 3%, 5%) on the quality, bacterial community and antioxidant capacity of alfalfa silage. The results showed that 5% phycocyanin supplementation could maintain dry matter (DM), crude protein (CP) and water-soluble carbohydrate (WSC) content; increase lactic acid (LA) content; decrease pH and butyric acid (BA) and ammonia nitrogen (NH_3_-N) content; and improve fermentation quality. At the same time, the contents of total antioxidant capacity (TAOC), total phenol content (TP), polysaccharide content (P) and total flavonoid content (F) in the addition group were significantly increased, the antioxidant capacity was enhanced and the abundance of lactic acid bacteria was increased, which was positively correlated with silage quality. Phycocyanin can improve the metabolic function of carbohydrates and amino acids and promote the production of secondary metabolites. The application of phycocyanin broadens the variety of additives for alfalfa silage.

## 1. Introduction

Alfalfa (*Medicago sativa* L.) is widely cultivated in the world because of its excellent drought and cold tolerance, multiple-cutting tolerance, high yield and high nutritional value. The common processing and utilization applications of alfalfa include hay preparation and silage [1]. Alfalfa hay is easily affected by weather, transportation and storage during production, which leads to a serious loss of nutrients. In contrast, the silage method can effectively reduce the nutritional loss caused by these factors, and keep the green and juicy characteristics of alfalfa, so that it has excellent flavor and high nutritional value [2]. However, it is difficult to produce silage from alfalfa alone due to its high buffer energy value, low soluble carbohydrate content and small number of naturally attached lactic acid bacteria [3]. Therefore, when silage production from alfalfa is carried out, it is usually necessary to add some auxiliary materials to improve its fermentation quality.

At present, the use of natural and environmentally friendly additives to protect livestock health and improve production efficiency has become one of the hot spots in this research field [4]. As a key ingredient that can eliminate free radicals and reduce oxidative pressure, antioxidants are widely used in animal feed and are essential additives [5]. Some nutrients in feed (such as vitamin A, vitamin E, unsaturated fatty acids, etc.) are easily oxidized during storage, and antioxidants can slow down the oxidation process of fats and other ingredients [6], thereby extending the shelf life of feed and maintaining its nutritional value and taste [7]. At the same time, antioxidants can help maintain the immune system function of animals, improve disease resistance and promote growth and production performance [8]. Certain antioxidants can also fight the growth of mold, thereby reducing the production of mycotoxins in feed and protecting the health of animals [9]. Silage, as the main coarse feed in the ruminant diet, accounts for more than half of their diet. If the antioxidant properties of silage can be enhanced, then it can become a functional forage product with antioxidant properties, thereby helping to not only improve the health and performance of animals, but also enhance the antioxidant capacity of animals and their products [10].

Phycocyanin is a water-soluble pigment protein extracted from prokaryotes such as cyanobacteria and has significant antioxidant properties. Its antioxidant capacity has been shown in some studies to be superior to that of other common antioxidants, and natural antioxidants are generally more compatible with the body and have fewer side effects than synthetic antioxidants. Among them, phycocyanin has outstanding free radical scavenging ability [11]. Regarding the main sources of oxidative stress, such as superoxide anion free radicals and hydroxyl free radicals, phycocyanin can effectively remove them to avoid damaging the cell membrane and DNA, thus preventing cell aging and disease [12]. At the same time, phycocyanin can inhibit lipid peroxidation and reduce the production of lipid peroxidation, thus protecting the integrity of the cell membrane and preventing cell damage. In addition, phycocyanin can also increase the activity of antioxidant enzymes in animals, such as malondialdehyde (MDA), superoxide dismutase (SOD), catalase (CAT), and peroxidase (POD), which play a role in scavenging free radicals and reducing oxidative stress in the body. Finally, phycocyanin can enhance the antioxidant defense system in cells, protect cells from oxidative damage and maintain the normal function of cells [13]. Antioxidants can improve oxygen management during the production of silage and reduce the occurrence of oxidation reactions. This helps to create an environment that is more conducive to the growth of anaerobic microorganisms and promotes the activity of beneficial microorganisms such as lactic acid bacteria, thereby improving the fermentation efficiency of silage.

At present, there are few studies on phycocyanin in the silage field, and there are still gaps in the research on its production and application. In conclusion, phycocyanin as a silage additive has the potential to improve the fermentation quality and antioxidant properties of alfalfa silage. The effects of phycocyanin supplementation on the quality, microbial composition and antioxidant activity of alfalfa silage were studied in order to provide a scientific basis and practical reference for preparing high-quality alfalfa silage and developing functional feed products.

## 2. Materials and Methods

### 2.1. Source of Experimental Materials

Alfalfa (WL319) was harvested at the third cutting during the early flowering stage from Liutu Village, Tumed Left Banner, Hohhot, Inner Mongolia Autonomous Region, at the Inner Mongolia Agricultural University Science and Technology Park. The stubble height was 8–10 cm, and the alfalfa was chopped to 2–3 cm lengths. Phycocyanin powder was purchased from Inner Mongolia Yike Biotechnology Co., Ltd. (Ordos, Inner Mongolia Autonomous Region, China).

### 2.2. Silage Preparation

In this study, one control (CK) group and three experimental groups (D1, D3 and D5 were set up. The control group was fed 400 g of alfalfa silage without the phycobilin additive, and the experimental groups were supplemented with phycobiliprotein additives equivalent to 1%, 3% and 5% of the weight of the control group, respectively. The water content of alfalfa silage was 60%, and the raw material was cut into 2 cm samples and sealed in a special silage bag under vacuum. The fermentation process was carried out at room temperature (25 °C), and there were 3 replicates in each treatment group. The fermentation process lasted for 60 days, at the end of which the samples were analyzed to obtain a series of comparative physicochemical and nutrient indices.

### 2.3. Component Analysis

The samples underwent a drying process for 72 h at 65 °C to determine their dry matter (DM) content. Crude protein (CP) was analyzed using the Dumas nitrogen determination method with a Dumas-01 analyzer from Gerhardt Analytical Instruments Co., Ltd. (Guangzhou, China). For the quantification of acid detergent fiber (ADF) and neutral detergent fiber (NDF), an ANKOM fiber analyzer (Model: A2000i) from Beijing ANKOM Technology Co., Ltd., Beijing, China, was employed. Water-soluble carbohydrates (WSC) were assessed using anthrone colorimetry [14]. pH levels were measured with an acidity meter from Shanghai Yida Scientific Instruments Co., Ltd., Shanghai, China, (Model: LEICI pH S-3C) in China. The identification of lactic acid (LA), acetic acid (AA), propionic acid (PA) and butyric acid (BA) was conducted using high-performance liquid chromatography with a Waters e2695 module from Milford, MA, USA. Ammonia nitrogen (NH_3_-N) content was determined using the phenol–hypochlorite colorimetric technique [15]. The total antioxidant capacity (TAOC) was determined using an improved ABTS method [16]. The determination of total phenolic (TP) content was carried out using the Folin–Ciocalteu colorimetric method improved by Cai et al. [17]. The polysaccharide (P) content was detected using the anthrone sulfuric acid colorimetric method [18]. The determination of total flavonoid content (F) was carried out using the colorimetric method improved by Chun et al. [19].

### 2.4. Microbial Sequencing

After the silage was opened, aseptic tube sampling was used to extract genomic DNA from the silage samples using the CTAB method, and then, the purity and concentration of the DNA were detected by agar gel electrophoresis. An appropriate amount of sample DNA was added to a centrifuge tube and the sample was diluted to 1 ng/μL with sterile water. The diluted genomic DNA was used as a template for PCR amplification of the 16S rDNA gene’s V3-V4 region with barcode-specific primers (515F: 5′-CCTAYGGGRBGCASCAG-3′ and 806R: 5′-GGACTACNNGGGTATCTAAT-3′). The PCR products were detected using 2% agarose gel electrophoresis. The DNA library was constructed using an NEBNext^®^ Ultra™ II DNA Library Prep Kit (Beijing, China). After library quality confirmation, sequencing was performed on a NovaSeq 6000 platform. The resulting amplicon sequence variants (ASVs) were aligned with the database using the classify-sklearn module in QIIME 2 to obtain species-level information for each ASV [20,21].

### 2.5. Statistical Analysis

In this study, an analysis of variance (ANOVA) with 3 replicates per group was performed using SPSS 26. Statistical significance was defined as *p*-values less than 0.05. The reliability of the sample means was evaluated using the standard error of the mean (SEM). Microsoft Excel 2010 was employed for table creation, while graph generation was conducted using Origin 2021 and R 4.1.2.

## 3. Results

### 3.1. Fresh Characteristics

The chemical composition and antioxidant activity of fresh alfalfa are shown in Table 1. The DM content of alfalfa was 28.60%FW, the CP content was 22.48%DM, the NDF content was 38.49%DM, the ADF content was 27.61%DM and the WSC content was 2.65%DM. The TAOC of alfalfa was 0.26%, the TP content was 0.73%, the P content was 1.04% and the F content was 1.21%.

### 3.2. Effects of Phycocyanin Additives on Chemical Composition

The chemical composition of silage is shown in Table 2. The phycocyanin additives had significant effects on the contents of DM, CP, NDF, ADF and WSC in silage (*p* < 0.05). The DM content in the D5 group was significantly higher than that in the other groups (*p* < 0.05). The CP content in the D5 group was significantly higher than that in the other groups, and in the CK group was the lowest (*p* < 0.05). The NDF content in the D5 group was significantly lower than that in the other groups, and in the CK group was the highest (*p* < 0.05). The ADF content in the D3 and D5 groups was significantly lower than that in the other groups, and in the CK group was the highest (*p* < 0.05). The WSC content in the D3 group was significantly higher than that in the other groups, and in the CK group was the lowest (*p* < 0.05).

### 3.3. Effects of Phycocyanin Additives on Fermentation Composition

The fermentation composition of silage is shown in Table 3. The phycocyanin additives had significant effects on the contents of pH, LA, AA, PA, BA and NH3-N in silage (*p* < 0.05). The pH in the D5 group was significantly lower than that in the other groups, and in the CK group was the highest (*p* < 0.05). The LA content in the D5 group was significantly higher than that in the other groups, and in the CK group was the lowest (*p* < 0.05). The AA content in the D3 and D5 groups was significantly lower than that in the other groups, and in the CK group was the highest (*p* < 0.05). The PA content in the D5 group was significantly lower than that in the other groups, and in the CK group was the highest (*p* < 0.05). The BA content in the CK group was significantly higher than that in the other groups, and in the D5 group was the lowest (*p* < 0.05). The NH3-N content in the CK group was significantly higher than that in the other groups, and in the D5 group was the lowest (*p* < 0.05).

### 3.4. Effects of Phycocyanin Additives on Antioxidant Activity

**Figure 1:**
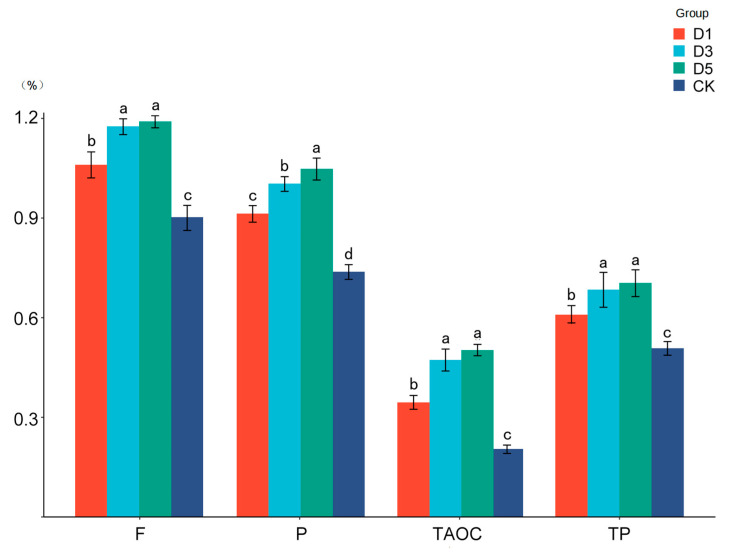
Antioxidant activity of alfalfa silage. CK, no additive control; D1, phycocyanin was added at 1%; D3, phycocyanin was added at 3%; D5, phycocyanin was added at 5%; Different lowercase letters indicate significant differences between treatments. TAOC, total antioxidant capacity (%); TP, total phenols; P, polysaccharides; F, flavonoid.

The antioxidant activity of silage is shown in Figure 1. The phycocyanin additives had significant effects on the contents of TAOC, TP, P and F in silage (*p* < 0.05). The TAOC in the D3 and D5 groups was significantly higher than that in the other groups, and in the CK group was the lowest (*p* < 0.05). The *TP content* in the D3 and D5 groups was significantly higher than that in the other groups, and in the CK group was the lowest (*p* < 0.05). The P content in the D3 group was significantly higher than that in the other groups, and in the CK group was the lowest (*p* < 0.05). The F content in the D3 and D5 groups was significantly higher than that in the other groups, and in the CK group was the lowest (*p* < 0.05).

### 3.5. Effects of Phycocyanin Additives on Microbial Community

The difference in alpha diversity in silage is shown in Table 4. The phycocyanin additives had significant effects on the Sobs (the number of species actually observed), Chao (an index to estimate species richness, where higher indices indicate greater species richness), Shannon (an indicator of species diversity in a biome, where the higher the index, the greater the diversity), Simpson (an indicator of the species uniformity of a biome, where smaller values indicate greater species diversity), and ACE (an indicator that estimates unobserved species richness, with higher indices indicating greater species richness) indices in silage (*p* < 0.05). The Sobs index in the D5 group was significantly higher than that in the other groups, and in the CK group was the lowest (*p* < 0.05). The Chao index in the D5 group was significantly higher than that in the other groups, and in the CK group was the lowest (*p* < 0.05). The Shannon index in the D3 and D5 groups was significantly lower than that in the other groups, and in the CK group was the highest (*p* < 0.05). The Simpson index in the D3 and D5 groups was significantly higher than that in the other groups, and in the CK group was the lowest (*p* < 0.05). The ACE index in the D5 group was significantly higher than that in the other groups, and in the CK group was the lowest (*p* < 0.05).

**Figure 2:**
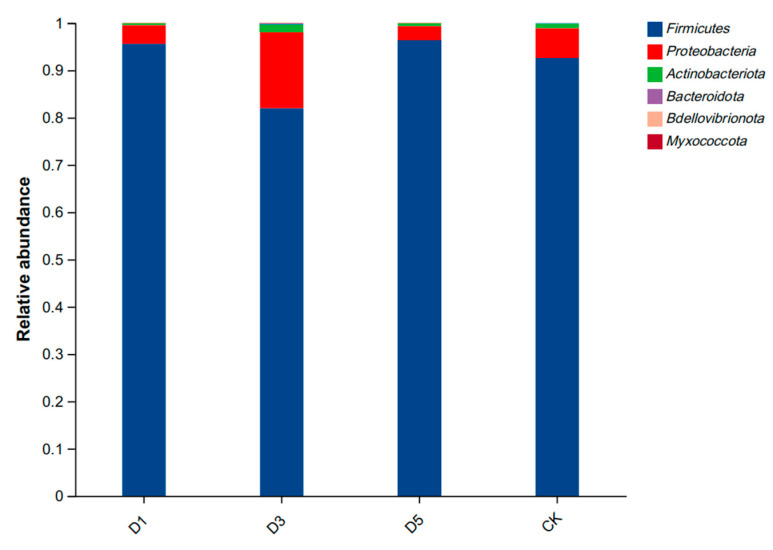
Bacterial phylum composition of alfalfa silage.

The composition of the bacterial phylum levels is shown in Figure 2. The dominant phyla in all groups were *Firmicutes* and *Proteobacteria*. The abundance of *Firmicutes* in the D1 and D5 groups was higher than that in the CK group, while the abundance of *Proteobacteria* was lower than that in the CK group. The abundance of *Firmicutes* in the D3 group was lower than that in the CK group, and *Proteobacteria* was higher than that in the CK group.

**Figure 3:**
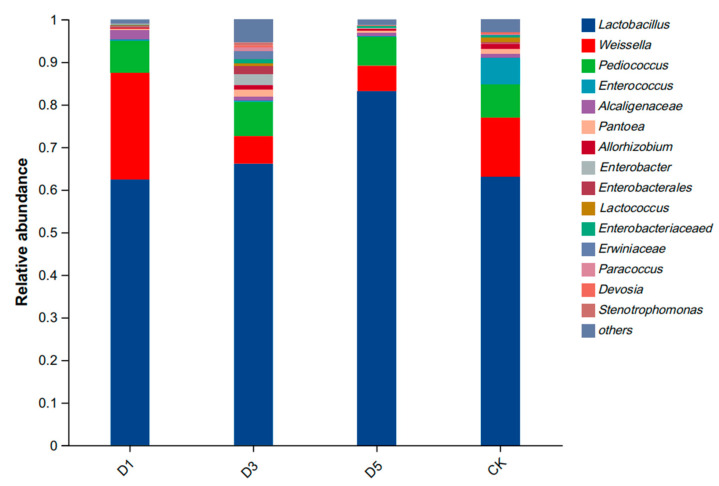
Bacterial phylum composition of alfalfa silage.

The composition of the bacterial genus levels is shown in Figure 3. The dominant genera in each group were *Lactobacillus*, *Weissella*, *Pediococcus* and *Enterococcus*. The abundance of *Lactobacillus* in the phycocyanin group was higher than that in the CK group, and the abundance in the D5 group was the highest. The D1 group had the highest abundance of *Weissella*, followed by the CK group, and the D3 and D5 groups had the lowest abundance. There was no difference in the abundance of *Pediococcus* among all groups. The abundance of *Enterococcus* in the CK group was higher than that in the other groups, while that in the phycocyanin group was low.

### 3.6. Functional Prediction of Bacterial Communities

**Figure 4:**
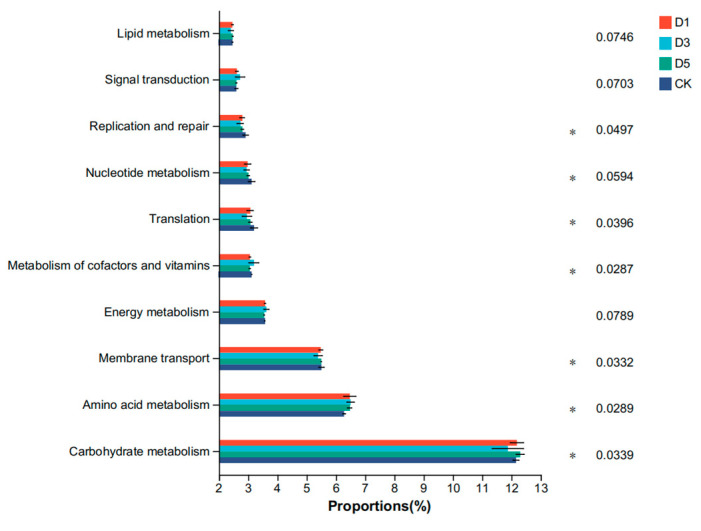
Functional prediction of bacteria at level 2. * *p* < 0.05.

**Figure 5:**
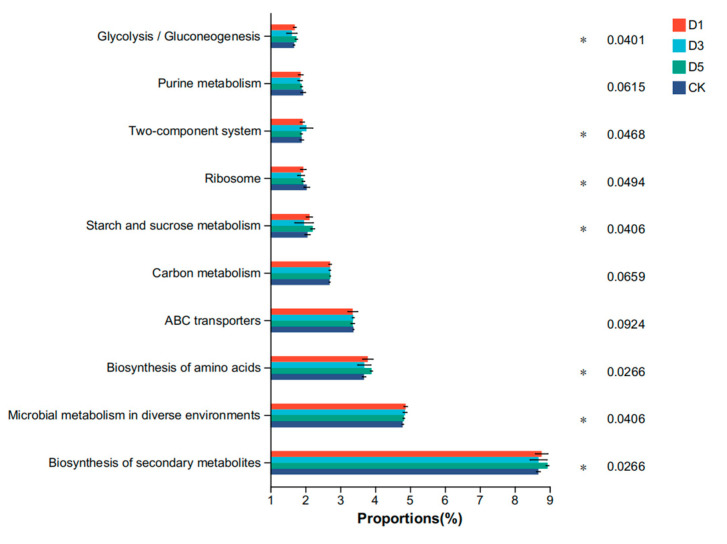
Functional prediction of bacteria at level 3. * *p* < 0.05.

The results based on the KEGG database at level 2 are shown in Figure 4. The main functions of each group were carbohydrate metabolism, amino acid metabolism, membrane transport, energy metabolism and the metabolism of cofactors and vitamins. The carbohydrate metabolism and amino acid metabolism of the D1 and D5 groups were significantly higher than those of the CK group (*p* < 0.05). The metabolism of cofactors and vitamins of the D3 group was significantly higher than that of the other groups (*p* < 0.05). The results based on the KEGG database at level 3 are shown in Figure 5. The main functions of each group were the biosynthesis of secondary metabolites, microbial metabolism in diverse environments, the biosynthesis of amino acids, abc transporters and carbon metabolism. The biosynthesis of secondary metabolites and biosynthesis of amino acids of the D1 and D5 groups were significantly higher than those in the CK group (*p* < 0.05). The microbial metabolism in diverse environments of the D3 group was significantly higher than that in the other groups (*p* < 0.05).

### 3.7. Correlation Between Silage Quality and Bacteria at Genus Level

**Figure 6:**
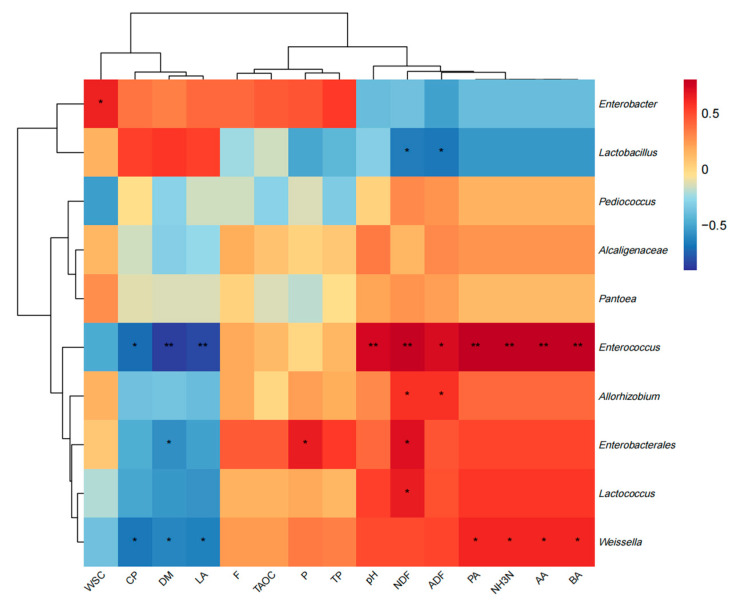
Heatmap of Pearson correlation between silage quality and bacteria. Red represents positive correlation, while blue represents negative correlation. Significance level is shown as follows: * *p* < 0.05, ** *p* < 0.01.

The correlations between silage quality and bacterial genus are shown in Figure 6. Lactobacillus was negatively correlated with NDF and ADF (*p* < 0.05). *Enterobacter* was positively correlated with WSC (*p* < 0.05). *Enterococcus* was extremely positively correlated with pH, NDF, AA, PA, BA and NH3-N (*p* < 0.01), positively correlated with ADF (*p* < 0.05), extremely negatively correlated with DM and LA (*p* < 0.01), and negatively correlated with CP (*p* < 0.05). *Allorhizobium* was positively correlated with NDF and ADF (*p* < 0.05). *Enterobacterales* was positively correlated with NDF and P (*p* < 0.05) and negatively correlated with DM (*p* < 0.05). *Lactococcus* was positively correlated with NDF (*p* < 0.05). *Weissella* was positively correlated with AA, PA, BA and NH3-N (*p* < 0.05) and negatively correlated with DM, CP and LA (*p* < 0.05).

## 4. Discussion

### 4.1. Effects of Phycocyanin Additive on Chemical Composition of Silages

The results of this study show that phycocyanin as an additive had a significant effect on the chemical composition of alfalfa silage. Especially at high concentrations, the DM and CP contents of the D5 group were significantly higher than those of the other treatment groups, while the CK group showed the lowest DM and CP contents. This indicates that high concentration of phycocyanin can effectively reduce the loss of nutrients during silage production and improve the nutritional value of silage. Dry matter is a key indicator to measure water loss and nutrient preservation during silage fermentation [22]. The high DM content in the D5 group may be due to the water absorption of phycocyanin molecules during silage production, thereby increasing the content of dry matter [23]. The crude protein content is directly related to the nutritional level of the feed. The increase in CP content in the D5 group may be due to the phycocyanin present in the antioxidant mechanism, reducing oxidative stress and protecting the activity of intracellular antioxidant enzymes, thereby reducing protein oxidation and degradation [24]. On the other hand, in the study of Akalin et al. [25] on the effect of *S. platensis* biomass on the microbial activity of traditional yogurt and probiotics during cold storage, the protein content of algae-containing samples was significantly increased compared with the control group. The addition of 5% phycocyanin in the D5 group was the highest addition in this experiment, and similar results were obtained [26]. At the same time, the significant decrease in NDF and ADF content in the D5 group indicated that phycocyanin also improved the fiber digestibility of alfalfa silage. NDF and ADF reflect the indigestible parts of the plant cell wall. Lower NDF and ADF content means that the feed is more easily digested and absorbed by livestock [27]. Therefore, this study shows that phycocyanin can not only retain nutrients in silage, but also reduce fiber content, thereby improving the overall quality of silage.

### 4.2. Effects of Phycocyanin Additive on Fermentation Quality of Silages

The changes in NH_3_-N concentration, organic acid content and pH value are the key indices used to evaluate the fermentation quality of silage [28]. The role of phycocyanin in improving the fermentation quality of alfalfa silage was particularly significant, especially in the performance of the D5 group. During the fermentation process, the NH_3_-N concentration and pH value of the D5 group decreased significantly, and the LA content increased significantly. This may be because the degradation of the fiber increases the content of WSC and promotes the production of lactic acid, thereby reducing the pH value. The decrease in NH_3_-N content in silage in the experimental group may be due to the inhibition of plant or bacterial proteolytic enzyme activity [29]. Similarly, apple pomace and grape pomace are also rich in polyphenols, such as phenolic acids and flavonoids [30,31]. They increase the lactic acid content in alfalfa silage during the silage production process, while reducing the pH value and NH_3_-N [32]. Higher AA and BA content usually means that poor fermentation occurs during silage production [33,34]; in particular, the reproduction of BA bacteria will lead to a decline in silage quality [35]. In this study, the content of AA and BA in the D5 group was significantly lower than that in the CK group, which may be due to the effect of the inhibition of phycocyanin on the reproduction of the microbiome, which is not conducive to silage fermentation [36], thereby reducing the content of AA and BA produced. On the other hand, it may be because the degradation of fiber increases the content of WSC, promotes the fermentation of lactic acid bacteria and increases the production of lactic acid, and thus inhibits the production of AA and BA. This result further supports the positive role of phycocyanin in optimizing the silage fermentation process. The above results show that phycocyanin not only promoted the production of lactic acid, but also reduced the accumulation of secondary fermentation products such as acetic acid and BA. Therefore, the improvement in the D5 group in this aspect means that phycocyanin can promote the formation of main fermentation products, reduce the accumulation of secondary and poor fermentation products and further improve the fermentation quality of silage.

### 4.3. Effects of Phycocyanin Additive on Antioxidant Properties of Silages

Antioxidants play a key role in alleviating oxidative stress, improving the quality of meat and dairy products and prolonging their shelf life. They not only help to maintain animal health, but also promote the improvement of production performance [8], increase the milk production of dairy cows, improve the daily weight gain of beef cattle and enhance animals’ overall immune function [37]. In terms of silage additives, antioxidants can improve the fermentation process of silage, inhibit the growth of harmful microorganisms and reduce oxidative stress during fermentation, thereby improving the quality of silage and further enhancing the stability and preservation of silage [38]. This study showed that in terms of antioxidant activity, the addition of phycocyanin showed a significant positive effect; in particular, the TAOC of the D3 and D5 groups was significantly higher than that of the other treatment groups, while the antioxidant activity of the CK group was the lowest, which may be related to the antioxidant capacity of phycocyanin. This result is consistent with the study of Bermejo P et al. [39]. They found that phycocyanin has strong free radical scavenging ability and can inhibit the formation of peroxides, thereby reducing the loss of nutrients caused by oxidation reactions. In addition, the D3 and D5 groups also showed significant increases in TP, P and F. The enrichment of these components in silage further enhanced their antioxidant properties. As natural antioxidants, phenolic compounds and flavonoids can slow down the process of oxidation reactions by scavenging free radicals [40], which may further prevent the degradation of fatty acids and proteins in silage. Therefore, phycocyanin further enhanced the overall antioxidant capacity of silage by promoting the increase in these antioxidant components. The improvement of antioxidant activity by phycocyanin not only helps to maintain the nutritional stability of feed [11], but also may improve the preservation effect of silage during long-term storage. Therefore, phycocyanin has important application value in the antioxidant protection of silage, and in feed with a long storage period, this advantage may be especially prominent.

### 4.4. Effect of Phycocyanin Additive on Microorganisms of Silage

*Lactobacillus* plays an important role in the fermentation process of silage [41]. The addition of phycocyanin significantly changed the microbial community structure in silage, especially in the D5 group. The abundance of Lactobacillus was significantly higher in this group than in the other groups, while the abundance of *Enterococcus* was the highest in the CK group. This may be because phycocyanin has antioxidant, anti-inflammatory and antibacterial activities. These active ingredients can enhance the metabolic activity of lactic acid bacteria [42], and inhibit putrefying bacteria [36], so that the abundance of lactic acid bacteria is increased, which helps to increase the production of lactic acid and rapidly reduce the pH value, thereby inhibiting the growth of harmful bacteria. On the other hand, phycocyanin may promote the accumulation of phenolic compounds and flavonoids during silage production. These compounds promote the fermentation of lactic acid bacteria during silage production, thereby increasing the lactic acid content in silage alfalfa [32]. Therefore, the high abundance of lactic acid bacteria in the D5 group further proved that phycocyanin had a positive effect on silage fermentation. This is consistent with the results of previous fermentation quality studies, indicating that phycocyanin helps to enhance the activity of beneficial bacteria and optimize the silage fermentation process. In contrast, the high abundance of *Enterococcus* and *Weissella* in the CK group showed that the reproduction of putrefying bacteria in the silage was more vigorous without the addition of phycocyanin. The negative correlation between *Enterococcus* and silage quality is consistent with the study of Sun et al. [43]. They pointed out that a high abundance of *Enterococcus* was positively correlated with inferior fermentation indices such as NDF, ADF and NH_3_-N during fermentation, but negatively correlated with lactic acid production. The significant decrease in the abundance of *Enterococcus* in the D5 group may be due to the fact that phycocyanin effectively inhibits the reproduction of these unfavorable microbial communities through the antioxidant mechanism, reduces the adverse fermentation phenomenon in silage, and thus improves the quality of the feed. This result is consistent with Chandra P et al. [44]. They found that the antioxidants produced by microorganisms have the mechanism of inhibiting harmful microorganisms. In summary, phycocyanin changed the microbial community structure in silage through an antioxidant mechanism; in particular, it increased the abundance of lactic acid bacteria, inhibited the reproduction of putrefying bacteria and significantly improved the fermentation quality and nutritional value of silage. This provides a new scientific basis for the application of phycocyanin in silage.

### 4.5. Correlation Analysis of Effects of Phycocyanin Additives on Silage Quality and Bacterial Genus Level

Through the correlation analysis of silage quality and bacterial genus level, the effect of microbial community on silage fermentation quality was further revealed. The results showed that *Lactobacillus* was significantly negatively correlated with NDF and ADF (*p* < 0.05), which meant that with an increase in *Lactobacillus* abundance, the fiber content in silage decreased and the fiber digestibility increased. This is consistent with the research results of Zhu et al. [45]. They found that the high abundance of lactic acid bacteria can improve the fermentation quality of silage, reduce the fiber content, and then improve the digestibility of silage. Mu et al. [46] also reached a similar conclusion, pointing out that the increase in the abundance of lactic acid bacteria in silage supplemented with lactic acid bacteria helps to accelerate the degradation of fibers and significantly improve the digestibility of NDF and ADF. In addition, *Enterococcus* showed a significant positive correlation with multiple adverse fermentation indicators, such as pH, NDF, NH_3_-N and BA (*p* < 0.05), indicating that a high abundance of *Enterococcus* was closely related to a decline in silage quality, especially related to protein degradation and the accumulation of adverse fermentation products. This is consistent with the study of Sun et al. [43]. They pointed out that a high abundance of *Enterococcus* was positively correlated with a poor fermentation index in silage, which further supported its adverse effects on silage fermentation. Similarly, Liu B et al. [47] found that a high abundance of *Enterococcus* hindered the production of lactic acid and was significantly correlated with a decrease in lactic acid content in silage. *Weissella* showed a positive correlation with poor fermentation indicators, including NH_3_-N, AA and BA (*p* < 0.05), and a negative correlation with dry matter, crude protein and lactic acid (*p* < 0.05). Cai Y et al. [48] pointed out that *Weissella* was dominant in the early stage of silage fermentation, but its abundance gradually decreased with the proliferation of lactic acid bacteria, which helped to improve the fermentation quality of silage. Zhu et al. [40] also found that *Weissella* was closely related to the formation of bad fermentation products in silage, especially in the absence of lactic acid bacteria, and an increase in its abundance would aggravate the deterioration of silage quality. In addition, other bacterial genera such as *Enterobacterales* and *Lactococcus* were also significantly correlated with some fermentation indicators. Zhao S et al. [49] pointed out that *Enterobacteriaceae* was positively correlated with NH_3_-N and BA production in silage, while *Lactococcus* was positively correlated with NDF content, indicating that the genus may hinder the degradation of fiber in some cases, thereby affecting the digestibility of feed. In summary, the effects of different bacterial genera on the fermentation process of silage are complex. An increase in lactic acid bacteria is closely related to the improvement of fermentation quality, while a high abundance of *Enterococcus* and *Weissella* is related to the accumulation of undesirable fermentation products in silage. Our results provide a new scientific basis for optimizing silage fermentation quality by regulating the microbial community.

### 4.6. Functional Prediction of Phycocyanin Additive’s Effect on Silage Microorganisms

Based on the functional prediction results of the KEGG database, the addition of phycocyanin significantly changed the microbial community structure of alfalfa silage and enhanced the metabolic function of microorganisms. Specifically, the carbohydrate metabolism and amino acid metabolism levels of the D1 and D5 groups were significantly higher than those of the CK group. Carbohydrate and amino acid metabolism are two crucial metabolic pathways in the process of microbial fermentation. Enhancing these metabolic activities shows that phycocyanin can promote the utilization of nutrients in feed by microorganisms, thereby improving the fermentation quality and nutritional value of feed. Zhao M et al. [50] showed that the high metabolic activity of lactic acid bacteria helps to improve the utilization of carbohydrates and accelerate the production of metabolites, thereby improving the fermentation quality and stability of silage. The biosynthesis of secondary metabolites and amino acids in the D1 and D5 groups was also significantly enhanced, further indicating that phycocyanin promoted the production of beneficial metabolites. Wu G. [51] found that amino acid metabolism is not only an important supplement to carbohydrate metabolism, but also further improves the nutritional value of feed by promoting the production of secondary metabolites. These secondary metabolites are of great significance for the flavor, stability and storage time of silage, especially in prolonging the storage period of silage. These products help to inhibit the growth of harmful microorganisms and delay the deterioration of feed quality, which is consistent with the results of this study. The functions of ‘microbial metabolism in diverse environments’ and ‘biosynthesis of amino acids’ in the KEGG three-level functional classification of the D5 group were significantly higher than those of the control group. This indicated that phycocyanin promoted the efficient utilization of nutrients by microorganisms in complex environments by enhancing the metabolic capacity of microorganisms, and further improved the quality of silage. Kumar V et al. [52] showed that the metabolic capacity of microorganisms is essential for nutrient metabolism during silage fermentation, and the production of secondary metabolites contributes to the overall stability and efficiency of silage fermentation. In summary, phycocyanin can significantly improve the metabolism of carbohydrates and amino acids of microorganisms and promote the production of secondary metabolites, and thus significantly improve silage fermentation efficiency and feed’s nutritional value. This provides an important scientific basis for the application of phycocyanin in silage, especially in improving the quality of silage.

## 5. Conclusions

This study revealed the effects of phycocyanin supplementation on the quality, bacterial community and antioxidant capacity of alfalfa silage. The results showed that 5% phycocyanin supplementation could maintain dry matter (DM), crude protein (CP) and water-soluble carbohydrate (WSC) content; increase lactic acid (LA) content; decrease pH and butyric acid (BA) and ammonia nitrogen (NH_3_-N) content; and improve fermentation quality. At the same time, the total antioxidant capacity (TAOC), total phenol (TP) content, polysaccharide content (P) and total flavonoid content (F) in the addition group were significantly increased, the antioxidant capacity was enhanced and the abundance of lactic acid bacteria was increased, which was positively correlated with silage quality. Phycocyanin can improve the metabolic function of carbohydrates and amino acids and promote the production of secondary metabolites. The application of phycocyanin broadens the variety of additives for alfalfa silage.

## Figures and Tables

**Table 1:** Chemical compositions and antioxidant activity of fresh alfalfa.

	Items	Alfalfa	SEM
Chemical composition	DM (%FW)	28.60	1.112
	CP (%DM)	22.48	0.293
	NDF (%DM)	38.49	0.445
	ADF (%DM)	27.61	0.505
	WSC (%DM)	2.65	0.185
Antioxidant activity	TAOC (%)	0.26	0.009
	TP (%)	0.73	0.011
	P (%)	1.04	0.010
	F (%)	1.21	0.012

FW, fresh weight; DM, dry matter; CP, crude protein; NDF, neutral detergent fiber; ADF, acid detergent fiber; WSC, water-soluble carbohydrate; TAOC, total antioxidant capacity (%); TP, total phenols; P, polysaccharides; F, flavonoid.

**Table 2:** Chemical composition of alfalfa silage.

Items	Treatment				SEM
	CK	D1	D3	D5	
DM (%FW)	37.99 b	41.03 b	42.98 b	43.88 a	0.596
CP (%DM)	20.77 d	21.10 c	21.69 b	23.49 a	0.234
NDF (%DM)	42.96 a	40.66 b	39.50 b	37.28 c	0.564
ADF (%DM)	39.41 a	37.68 ab	36.51 bc	35.01 c	0.502
WSC (%DM)	1.63 b	2.05 ab	2.23 a	2.02 ab	0.097

FW, fresh weight; DM, dry matter; CP, crude protein; NDF, neutral detergent fiber; ADF, acid detergent fiber; WSC, water-soluble carbohydrates; CK, no additive control; D1, phycocyanin was added at 1%; D3, phycocyanin was added at 3%; D5, phycocyanin was added at 5%; SEM, standard error of the mean. Different lowercase letters indicate significant differences between treatments; *p* < 0.05.

**Table 3:** Fermentation composition of alfalfa silage.

Items	Treatment				SEM	*p* Value
	CK	D1	D3	D5		
pH	4.66 a	4.51 b	4.39 c	4.27 d	0.039	<0.05
LA (%DM)	3.29 c	3.78 b	3.82 b	4.17 a	0.055	<0.05
AA (%DM)	1.13 a	0.85 b	0.77 c	0.75 c	0.074	<0.05
PA (%DM)	0.43 a	0.35 b	0.31 b	0.25 c	0.117	<0.05
BA (%DM)	0.12 a	0.09 b	0.08 b	0.06 b	0.001	<0.05
NH3-N (%TN)	0.48 a	0.43 b	0.42 b	0.40 b	0.017	<0.05

LA, lactic acid; AA, acetic acid; PA, propionic acid; BA, butyric acid; NH3-N, ammonia nitrogen; CK, no additive control; D1, phycocyanin was added at 1%; D3, phycocyanin was added at 3%; D5, phycocyanin was added at 5%; SEM, standard error of the mean. Different lowercase letters indicate significant differences between treatments.

**Table 4:** Bacterial α diversity of alfalfa silage.

Items	Treatment				SEM	*p* Value
	CK	D1	D3	D5		
Sobs	192.67 d	229.00 b	237.00 b	256.67 a	0.039	<0.05
Chao	196.77 d	229.34 b	239.69 b	257.91 a	17.616	<0.05
Shannon	1.92 a	1.78 b	1.53 c	1.48 c	0.119	<0.05
Simpson	0.36 c	0.44 b	0.59 a	0.61 a	0.041	<0.05
ACE	200.23 d	230.40 c	242.53 b	261.08 a	17.636	<0.05
Coverage	0.99	0.99	0.99	0.99	-	-

CK, no additive control; D1, phycocyanin was added at 1%; D3, phycocyanin was added at 3%; D5, phycocyanin was added at 5%; SEM, standard error of the mean. Different lowercase letters indicate significant differences between treatments.

## Data Availability

The raw sequence data were uploaded to the NCBI archive of sequence reads under the study record number PRJNA1175891.
